# Exploring inequalities in life expectancy and lifespan variation by race/ethnicity and urbanicity in the United States: 1990 to 2019

**DOI:** 10.1016/j.ssmph.2022.101230

**Published:** 2022-09-13

**Authors:** Isabel P. De Ramos, Amy H. Auchincloss, Usama Bilal

**Affiliations:** a Urban Health Collaborative, Drexel Dornsife School of Public Health, Philadelphia, PA, USA; b Department of Epidemiology and Biostatistics, Drexel Dornsife School of Public Health, Philadelphia, PA, USA

**Keywords:** Life expectancy, Lifespan variation, Inequalities, Race/ethnicity, Urbanicity, United States

## Abstract

**Background/Objective:**

Investigating trends in life expectancy and lifespan variation can highlight disproportionate mortality burdens among population subgroups. We examined inequalities in life expectancy and lifespan variation by race/ethnicity and by urbanicity in the US from 1990 to 2019.

**Methods:**

Using vital registration data for 322.0 million people in 3,141 counties from the National Center for Health Statistics, we obtained life expectancy at birth and lifespan variation for 16 race/ethnicity-gender-urbanicity combinations in six 5-year periods (1990–1994 to 2015–2019). Race/ethnicity was categorized as Hispanic, and non-Hispanic White, Black, and Asian/Pacific Islander. Urbanicity was categorized as metropolitan vs nonmetropolitan areas, or in six further detailed categorizations. Life expectancy and lifespan variation (coefficient of variation) were computed using life tables.

**Results:**

In 2015–2019, residents in metropolitan areas had higher life expectancies than their nonmetropolitan counterparts (79.6 years compared to 77.0 years). The widest inequality in life expectancy occurred between Asian/Pacific Islander women and Black men, with a 17.7-year gap for residents in metropolitan areas and a 16.9-year gap for residents in nonmetropolitan areas. Nonmetropolitan areas had greater dispersion around average age at death. Black individuals had the highest lifespan variations in both metropolitan and nonmetropolitan areas. Until the mid-2010s, life expectancy increased while lifespan variation decreased; however, recent trends show stagnation in life expectancy and increases in lifespan variation. Metropolitan-nonmetropolitan inequalities in both life expectancy and lifespan variation widened over time.

**Conclusion:**

Despite previous improvements in longevity, life expectancy is now stagnating while lifespan variation is increasing. Our results highlight that early-life deaths (i.e., young- and middle-age mortality) disproportionately affect Black individuals, who not only live the shortest lifespans but also have the most variability with respect to age at death.

## Highlights

• We examined trends in life expectancy and relative lifespan variation by race/ethnicity, gender, and urbanicity in the US.
• We found stagnating life expectancy and increasing lifespan variation over the last decade.
• There has been a diverging trend of life expectancy between urban and rural areas.
• Black individuals had a double burden of mortality: shorter and more variable lifespans.

## 1 Introduction

### 1.1 Longevity in the United States

In the United States, life expectancy at birth has dramatically improved in the past several decades, increasing from 68.2 years in the 1950s to 78.8 years currently in 2019 (National Center for Health Statistics (NCHS), 2021a). However, longstanding racial/ethnic inequalities in longevity remain. For example, there is a wide gap in longevity between Black and non-Hispanic White individuals (Harper et al., 2014). On the other hand, Hispanic individuals tend to experience a mortality advantage known as the “Hispanic paradox” in which they often outlive their White counterparts despite disadvantaged socioeconomic statuses (Arias et al., 2020), and a similar situation exists for non-Hispanic Asian/Pacific Islander (NHAPI) individuals who have the highest life expectancy. However, these inequalities vary geographically, both by region of the country and by urbanicity (Murray et al., 2006).

Inequalities in longevity by urbanicity suggest a “rural penalty,” where individuals in rural (nonmetropolitan) regions have higher mortality, manifested by shorter life expectancies, than their urban (metropolitan) counterparts (Cosby et al., 2019; Cossman et al., 2010; Miller & Vasan, 2021). Prior work examined this using data from 1968 to 2004 (Cosby et al., 2019) and 1969 to 2009 (Singh & Siahpush, 2014) and found a widening of the “rural penalty” over time, likely due to technology and health-enhancing resources becoming concentrated in urban areas (Cosby et al., 2019), and economic insecurity increasing in rural areas (Knapp et al., 2019). Based on that prior work, the urban advantage has conferred approximately 2.4 more years of life compared to individuals living in nonmetropolitan areas (Singh & Siahpush, 2014).

### 1.2 Lifespan variation

However, examining life expectancy by race/ethnicity and urbanicity alone obscures inequalities in longevity within groups (Shi et al., 2022). Measures of lifespan variation capture this within-group variability in age at death and therefore heterogeneity around the timing of death (Aburto & van Raalte, 2018; Shi et al., 2022). Substantially higher lifespan variations can have multiple implications (Aburto & van Raalte, 2018). At the population level, greater lifespan variabilities may point to a lack of effectiveness of health and social policies, such as safety nets and social protection policies, intended to protect disadvantaged populations (van Raalte et al., 2018). Such increases in lifespan variation among population subgroups may also signify the extent to how certain populations are living increasingly heterogenous lives and help identify which populations may require more targeted public health interventions (van Raalte et al., 2012, 2018). For individuals, the combination of generally low life expectancy and high variability in timing of death indicates reduced security around survival expectations, which could afflict decisions around life course planning (van Raalte et al., 2018). Therefore, examining trends in both life expectancy and lifespan variation can not only signal uneven mortality patterns between populations and point to targets of interventions, but also provide better insights into future mortality phenomena.

### 1.3 Objective

The literature on inequalities in lifespan variation by race/ethnicity and urbanicity combined is extremely limited in the US. Several studies have analyzed lifespan variation in Europe (Aburto et al., 2020; Seaman et al., 2019; van Raalte et al., 2011, 2014), but, to our knowledge, only two studies have focused on the US, one using national data for 1975–2017 (Hiam et al., 2021) and another state data for 1959–2017 (Xu et al., 2021). To our knowledge, no study has examined lifespan variation by multiple categories of race/ethnicity, US race-urbanicity combinations, nor incorporated newer mortality data.

With this study, we aim to examine inequalities in life expectancy and lifespan variation by race/ethnicity, gender, and urbanicity in the US from 1990 to 2019. By concurrently describing both inequalities in the average lifespan and variations in the lifespan, we can obtain a more complete picture of the mortality landscape of the US than by examining them independently.

## 2 Methods

### 2.1 Study setting and sources

We obtained vital registration data from the National Center for Health Statistics (NCHS, US Centers for Disease Control and Prevention) on all death records for the continental US from 1990 to 2019 (National Center for Health Statistics, 2007, 2017). The death records include age, gender, race/ethnicity, and county of residence (National Center for Health Statistics (NCHS), 2021b). We also obtained bridged-race postcensal population estimates from the US Census Bureau to obtain population denominators by age-, gender-race/ethnicity-, county, and year (National Center for Health Statistics (NCHS)). A small number of county boundaries changed during 1990–2019 thus were merged, as indicated in Appendix Table 1.

### 2.2 Variables

Age was operationalized in 5-year age groups, with the 0–4 group disaggregated into 0–1 and 1–4, and with 85+ as the open-ended age group. We operationalized gender as man or woman. We operationalized race/ethnicity using four bridged race groups commonly used for trend analyses (Singh et al., 2017). The bridged race groups bridge the single-race option with the multiple-race option that was incorporated into vital registration records in the early 2000's (Liebler & Halpern-Manners, 2008). The following categories were used: Hispanic, and non-Hispanic for all other race/ethnicities: White, Black, and Asian/Pacific Islander (hereafter referred to simply as White, Black, and Asian/Pacific Islander). We did not include non-Hispanic American Indian/Alaskan Natives as a bridged race group due to potential mismatches between death certificates and their respective denominators of population counts (Harper et al., 2021). In addition, we adopted the NCHS guidelines in the calculations of mortality rates for Hispanic individuals, excluding Hispanic death and population counts for the following states and years: Louisiana for 1990; New Hampshire for 1990–1992, and Oklahoma for 1990–1996 (National Center for Health Statistics, 2022). This exclusion represents 0.4–0.8% of the total Hispanic US population in those years.

We also linked county of residence (for both deaths and population) to the 2013 NCHS Urban-Rural Classification Scheme to classify residence urbanicity (National Center for Health Statistics (NCHS)). We operationalized urbanicity using two approaches: a metropolitan vs. nonmetropolitan categorization, and a more detailed 6-level categorization that splits metropolitan into 4 groups (large central, large fringe, medium and small) and nonmetropolitan into 2 groups (micropolitan and noncore). See Appendix Table 2 for more details on the urban-rural classification scheme. We also conducted a sensitivity analysis using the 1990 NCHS Urban-Rural Classification Scheme.

### 2.3 Mortality measures

We used two sets of outcomes: longevity and lifespan variation. Longevity was measured using life expectancy at birth, a commonly used metric of mortality that estimates the average age of death for a newborn (born today) if current mortality patterns hold in the future (Preston, Heuveline, & Guillot, 2001). We also computed life expectancies and lifespan variation at older ages (10, 35, and 65) to assess how mortality at specific ages influences inequalities. Higher values signify longer longevity.

To capture variability in this lifespan, we computed lifespan variation, understood as the variability in ages around an average age of death or an “age window” over which deaths occur (van Raalte et al., 2018). Following previous work by others (Aburto et al., 2020; Aburto & van Raalte, 2018; Edwards & Tuljapurkar, 2005; Németh, 2017), we operationalized lifespan variation as a relative measure of inequality using the coefficient of variation defined as the standard deviation of the mean age at death divided by the mean age at death (i.e., life expectancy) for each given racial/ethnic-gender-urbanicity group. Higher values signify greater variability in the ages of death and smaller values signify a more homogenous expected age of death. We preferred to capture lifespan variation using a measure of relative inequality (coefficient of variation) rather than absolute inequality (i.e., variance, standard deviation) because when used in longevity analyses, measures of absolute inequality are often measured in units of time and only capture the pace of aging; however, measures of relative inequality, or in our case, coefficient of variation, capture both the pace and shape of aging (Aburto et al., 2020). We conducted a sensitivity analysis to examine how different our findings would be if measures of lifespan as an absolute inequality (e.g., standard deviation) were used.

### 2.4 Statistical analyses

We conducted this analysis in three steps. First, we computed life expectancy at birth, $e_{0}$, and relative lifespan variation, measured as the coefficient of variation, $CV_{0}$ for each group (16 race/ethnic, gender, and urbanicity combinations for the main analysis, and 48 combinations for the disaggregated urbanicity analysis) across the six 5-year pooled periods: 1990–1994, 1995–1999, 2000–2004, 2005–2009, 2010–2014, and 2015–2019. Life expectancy ($e_{x}$) estimates were obtained from standard life tables (Preston et al., 2001) using age-, sex-, race/ethnicity-, and urbanicity-specific mortality rates for a specific period (2015–2019). To obtain standard errors and associated 95% confidence intervals for life expectancy, we used the Camarda method (Camarda, 2021). To calculate relative lifespan variation, we used Shkolnikov & Andreev's methodology to estimate the standard deviation (Shkolnikov & Andreev, 2010), and then calculated $CV_{0}$ by dividing the standard deviation over life expectancy at that age. We also show, in sensitivity analyses, standard deviation as a measure of absolute lifespan variation.

Second, we calculated both absolute and relative inequalities for life expectancy, as small relative differences can often mask large absolute differences. Absolute inequalities were captured by subtracting the nonmetropolitan life expectancy of a given racial-gender group from the metropolitan life expectancy or lifespan variation for that same racial-gender group. Relative inequalities were measured by dividing the metropolitan life expectancy of a given racial-gender group by the nonmetropolitan life expectancy for that same racial-gender group.

Finally, we described all results starting with the 2015–2019 period, comparing the four racial/ethnic groups by gender and two urbanicity classes (metropolitan and nonmetropolitan), followed by a description of trends (1990–1994, 1995–1999, 2000–2004, 2005–2009, 2010–2014, and 2015–2019) and disaggregated urbanicity (large central, large fringe, medium and small, micropolitan and noncore).

All statistical analyses were performed using R Statistical Software Version 4.0. Code is available for download at https://github.com/isabelderamos/LE_LV_US_Inequalities.

## 3 Results

### 3.1 Life expectancy

**Fig. 1:**
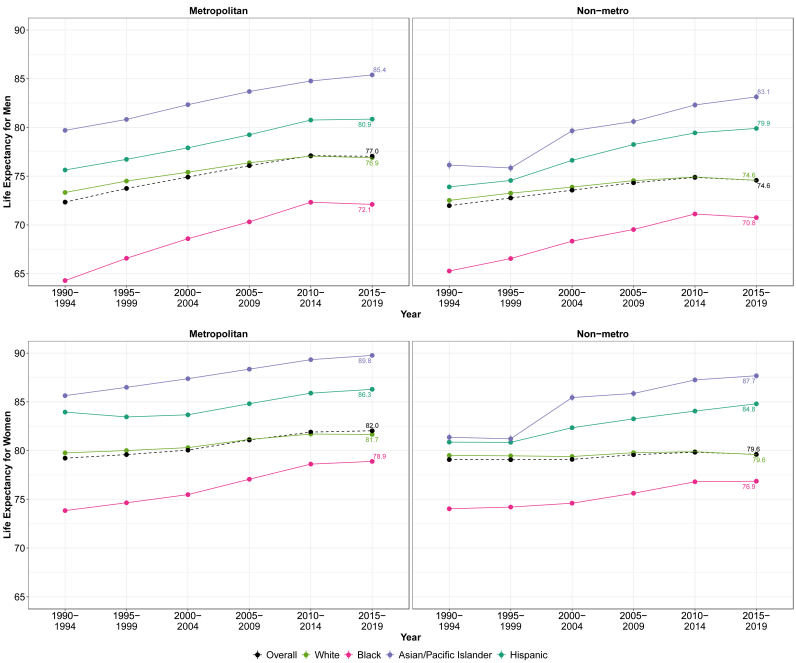
Temporal trends in life expectancy by race/ethnicity, gender, and urbanicity: United States, 1990–2019. Footnote: 95% confidence intervals are shown as error bars in life expectancy panels but may be difficult to see as some intervals are narrow. Refer to Appendix Table 5 for confidence interval values.

Appendix Table 4 provides a basic description of the 3,141 counties (37% in metro areas and 63% in non-metro) and 322.0 million people (86% in metro areas and 14% in non-metro, by 2015–2019) we included. Fig. 1 and Appendix Table 5 depict trends in life expectancy from 1990-1994 to 2015–2019 among White, Black, Asian/Pacific Islander, and Hispanic men and women by metropolitan category, while Table 1 displays the absolute and relative inequalities between metropolitan and nonmetropolitan individuals by race/ethnicity and gender for the last period (2015–2019). Across all racial-gender combination groups in 2015–2019 pooled, metropolitan individuals had higher life expectancies than their nonmetropolitan counterparts, and women had higher life expectancies than men in both metropolitan and nonmetropolitan areas. The magnitude of this gender differential was approximately 5 years in both metropolitan areas (82.0 vs 77.0 years) and nonmetropolitan areas (79.5 vs 74.5 years). Across racial groups, the widest life expectancy gap occurred between Asian/Pacific Islander women and Black men, with a metropolitan gap of 17.7 years (89.8 years vs 72.1 years) and a nonmetropolitan gap of 16.9 years (87.7 years vs 70.8 years). Within racial groups, White and Asian/Pacific Islander men and women had the widest absolute inequality by urbanicity, with White and Asian/Pacific Islander metropolitan men outliving their nonmetropolitan counterparts by 2.3 years and White and Asian/Pacific Islander metropolitan women outliving their nonmetropolitan counterparts by 2.1 years.

**Table 1:** Metropolitan-nonmetropolitan absolute and relative inequalities in life expectancy at birth and lifespan variation by race/ethnicity, gender, and urbanicity: United States, 2015–2019.

Race	Metropolitan-Nonmetropolitan Inequality in Life Expectancy			
	Men		Women	
	Absolute	Relative	Absolute	Relative
Overall	2.5	1.03	2.4	1.03
NHW	2.3	1.03	2.1	1.03
NHB	1.4	1.02	2.0	1.03
NHAPI	2.3	1.03	2.1	1.02
H	1.0	1.01	1.5	1.02

Footnote: Absolute differences are metro – nonmetro while relative differences are metro/nonmetro.

Generally, life expectancy increased monotonically over time for both metropolitan and nonmetropolitan men and women until the 2010–2014 period, after which life expectancy either changed minimally or slightly declined in both metropolitan and nonmetropolitan areas. There was one exception to this: White individuals in nonmetropolitan areas who experienced little change over the entire study period compared to the other racial/ethnic groups. Specifically, for nonmetropolitan White individuals, life expectancy increased from 1990-1994 to 2015–2019 by only 2.1 for men and only 0.1 years for women.

We also observed a convergence of life expectancies between metropolitan groups and a divergence of life expectancies between nonmetropolitan groups over time. In metropolitan areas, the difference between the highest (Asian/Pacific Islander) and lowest (Black) life expectancy narrowed, most notably for men, whose gap narrowed by 2.1 years (from a 15.4-year inequality in 1990–1994 to a 13.3-year inequality in 2015–2019) while that of women narrowed by 0.9 years (from a 11.8-year inequality in 1990–1994 to a 10.9-year inequality in 2015–2019). In nonmetropolitan areas, the difference between the highest (Asian/Pacific Islander) and lowest (Black) life expectancy diverged, widening by 1.5 years for men (from a 10.8-year inequality in 1990–1994 to a 12.3-year inequality in 2015–2019), and 3.4 years for women (from a 7.4-year disparity in 1990–1994 to a 10.8-year inequality in 2015–2019).

**Fig. 2:**
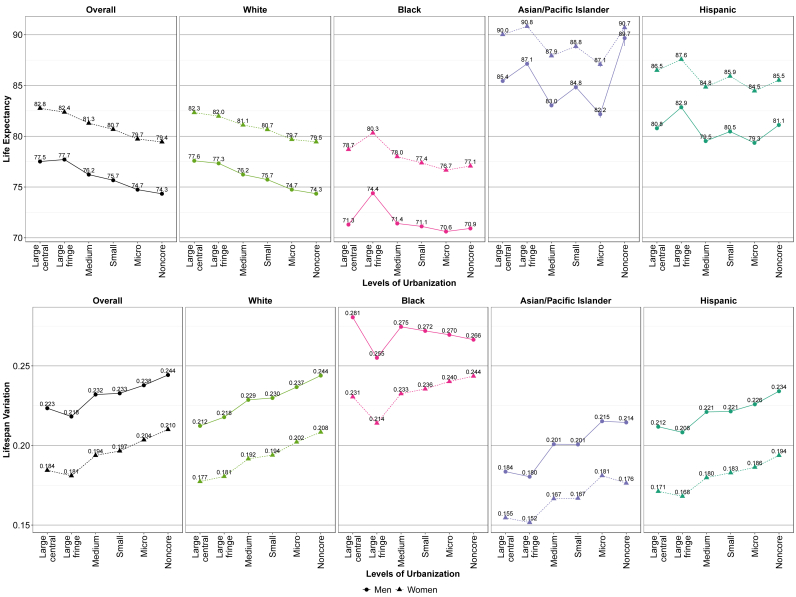
Life expectancy and lifespan variation (coefficient of variation) by race/ethnicity, gender, and levels of urbanization: United States, 2015–2019. Footnote: 95% confidence intervals are shown as error bars but may be difficult to see as some intervals are narrow. Refer to Appendix Table 5 for life expectancy confidence values.

Fig. 2 and Appendix Table 6 show life expectancies at birth by race/ethnicity, gender, and the disaggregated six levels of urbanization in the 5-year period of 2015–2019. Overall, we found a decreasing gradient in life expectancy across the continuum of urbanization, as life expectancy decreases as counties become less urban. This pattern was most evident among White men and women. Black men and women experienced this decreasing gradient in life expectancy except in the large fringe metropolitan county group, in which both racial/ethnic groups experienced their highest life expectancies. Asian/Pacific Islander men and women as well as Hispanic men and women experienced varying life expectancies across the urban-rural classification scheme without a clear gradient.

### 3.2 Lifespan variation

**Fig. 3:**
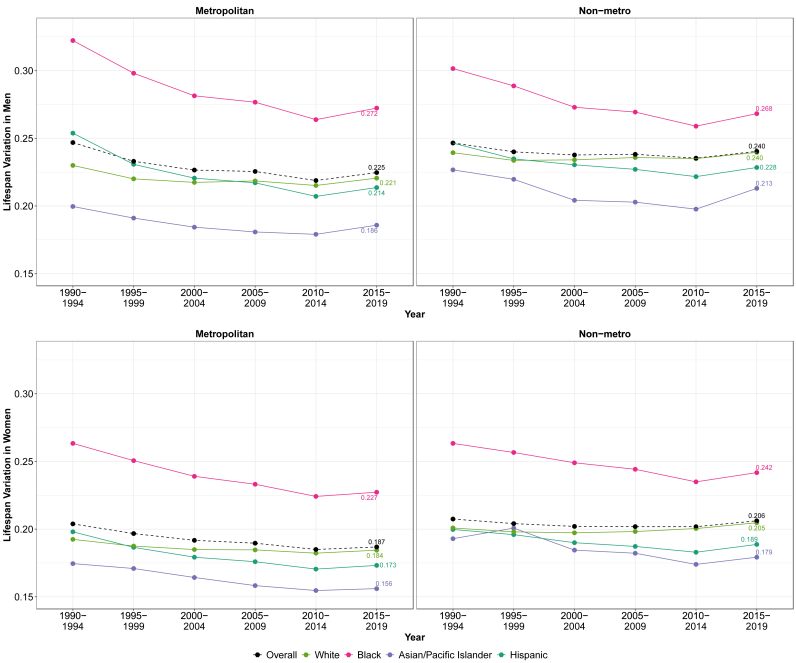
Temporal trends in lifespan variation by race/ethnicity, gender, and urbanicity: United States, 1990–2019.

Fig. 3 and Appendix Table 7 depict trends in relative lifespan variation (using the coefficient of variation) from 1990-1994 to 2015–2019 among White, Black, Asian/Pacific Islander, and Hispanic men and women by metropolitan category. Overall, and across most racial-gender combination groups, nonmetropolitan individuals experienced higher lifespan variations than their metropolitan counterparts, with the exception of Black men (i.e., metropolitan Black men saw higher variability around age at death). Overall, men had higher lifespan variations than women in both metropolitan and nonmetropolitan areas. Across racial/ethnic groups, the highest lifespan variations occurred among Black men and women in both metropolitan and nonmetropolitan areas. In fact, although metropolitan Black women outlive metropolitan White men by 2 years, metropolitan Black women had roughly the same if not greater variability around their age at death than White metropolitan men. In general, we observed a decrease in lifespan variation from 1990-1994 to 2005–2009 or 2010–2014, followed by a subsequent increase from 2010-2014 to 2015–2019.

Similar to life expectancy, the widest inequality in lifespan variation occurred between Black men and Asian/Pacific Islander women in both metropolitan and nonmetropolitan areas. Unlike trends in life expectancy, we observed a convergence of lifespan variations among men and women in both metropolitan and nonmetropolitan groups over time. Regardless of urbanicity, the difference between the highest (Asian/Pacific Islander) and lowest (Black) lifespan variation narrowed, most notably for men in metropolitan areas, whose gap in lifespan variability narrowed the most between 1990-1994 and 2015–2019. However, this convergence was mainly the result of lifespan variability improving among Black men and women in both metro and nonmetropolitan areas between 1990-1994 and 2010–2014.

Fig. 2 and Appendix Table 8 shows relative lifespan variation by race/ethnicity, gender, and the disaggregated six levels of urbanization in the 5-year period of 2015–2019. Overall, we found an increasing gradient in lifespan variation across the levels of urbanization, in which more metropolitan groups experienced lower lifespan variations and more nonmetropolitan groups saw higher lifespan variations, with a clearer gradient for White men and women. However, Black, Asian/Pacific Islander, and Hispanic men and women experienced their lowest lifespan variations in the large fringe metropolitan county group. One exception to the increasing gradient occurred among metropolitan Black men, in which lifespan variation decreased as areas became less urban (more rural).

### 3.3 Life expectancy and lifespan variation

**Fig. 4:**
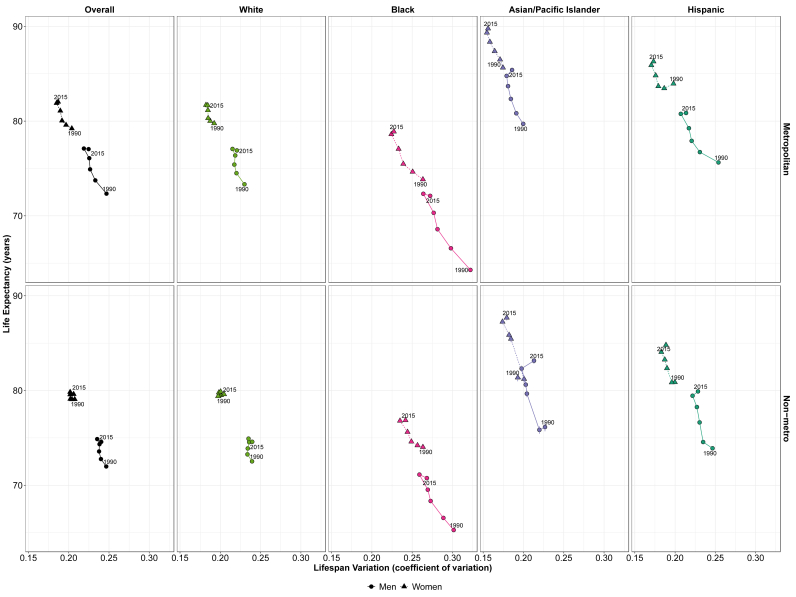
Association between life expectancy and lifespan variation (measured as coefficient of variation) by race/ethnicity, gender, and urbanicity: United States, 1990–2019. Footnote: 95% confidence intervals are shown as error bars but may be difficult to see as some intervals are narrow. Refer to Appendix Table 5 and for confidence interval values. Year shown refers to the first year in each period (e.g., 1990 corresponds to the 1990–1994 period).

Fig. 4 shows the correlation of life expectancy and relative lifespan variation (coefficient of variation) over time (values for 1990 and 2015 are labeled) by race/ethnicity, gender, and urbanicity. Overall, life expectancy and lifespan variation share an inverse relationship, where lower lifespan variations are correlated with higher life expectancies. Regardless of urbanicity and gender, most racial groups saw an increase in life expectancy from 1990-1994 to 2010–2014 (shown by the vertical increase towards the upper portion of the figure) and a decrease in lifespan variation (shown by the horizontal decrease towards the left). However, as shown in Fig. 3, from 2010-2014 to 2015–2019, all race-gender-urbanicity groups experienced an increase in lifespan variation.

### 3.4 Secondary analyses

Appendix Table 9 shows results for life expectancy at ages 10, 35 and 65 for the 2015–2019 period. In general, we observed a similar pattern to our main exploration of life expectancy and lifespan variation. At 10, 35, and 65 years, metropolitan men and women experienced higher life expectancies and lower lifespan variations than their nonmetropolitan counterparts. At each age, women continued to experience higher life expectancies and lower lifespan variations than men in metropolitan and nonmetropolitan areas. Importantly, regardless of urbanicity, life expectancy converged at older ages (35 and 65 years), suggesting substantial mortality differences below the age of 65. The widest inequality in life expectancy (still between Asian/Pacific Islander women and Black men) decreased from a 17.7- (at birth) to a 10.2-year (at 65 years) gap for residents in metropolitan areas and decreased from a 16.9- (at birth) to a 10.1-year (at 65 years) gap for residents in nonmetropolitan areas. We also documented a divergence of lifespan variation at older ages, with individuals at 35 and 65 years experiencing relatively large variations in timing of death relative to their remaining life expectancies.

Appendix Fig. 1 shows a comparison of a series of measures of lifespan variation (see Appendix Table 3 for definitions). For standard deviation and the coefficient of variation, our main measures of absolute and relative lifespan variation respectively, we found a very high correlation (r = 0.963). Furthermore, we conducted an additional analysis to examine how different the correlation between life expectancy and lifespan variation would be if lifespan variation was captured as a measure of absolute inequality (standard deviation) (Appendix Fig. 2). We found some differences related to the scale of inequality between measures of absolute and relative lifespan variation. For example, while overall relative and absolute lifespan variation of metropolitan men both decreased from 1990 to 2015, relative lifespan variation of nonmetropolitan men decreased from 1990 to 2015 and absolute lifespan variation increased. In Appendix Fig. 3 we compared both measures of lifespan variation against each other. We found an overall positive correlation between both measures, with higher relative lifespan variation correlated with higher absolute lifespan variation, especially in women. However, when exploring changes by period, we also found periods of negative correlation, especially in men. Specifically, from 1995-1999 to 2010–2014 we found decreasing relative lifespan variation with increasing absolute lifespan variation.

We also conducted a sensitivity analysis changing the urbanicity exposure from the 2013 NCHS Urban-Rural Classification Scheme to the 1990 definitions (Appendix Figs. 4 and 5). We found no appreciable differences for either life expectancy of lifespan variation, indicating that our results were robust to the choice of urbanicity exposure.

## 4 Discussion

### 4.1 Summary of findings

We examined trends in life expectancy and relative lifespan variation by race/ethnicity, gender, and urbanicity from 1990 to 2019 in the US. Regardless of urbanicity, life expectancy has increased from the 1990s until the mid 2010s, when life expectancy stagnated, and lifespan variation increased. We also found evidence of a widening rural penalty in life expectancy, with lower life expectancies for individuals living in nonmetropolitan areas compared to metropolitan areas. However, this overall pattern is heterogeneous by race/ethnicity and gender. Last, racial/ethnic inequalities in lifespan variation in addition to longevity show that Black individuals are consistently subjected to the “double burden of mortality” of lower life expectancies and higher lifespan variations.

### 4.2 Stagnating life expectancy and increasing lifespan variation

Life expectancy increased from the 1990s until the mid-2010's, when life expectancy stagnated for metropolitan areas and decreased for nonmetropolitan areas. Concurrently, lifespan variation increased for both metropolitan and nonmetropolitan areas during this last period. Examining life expectancy and lifespan variation at birth, 10-, 35- and 65-years of age reflected a convergence of life expectancy at older ages and a divergence of lifespan variation at older ages. According to Aburto et al. (2020), life expectancy and lifespan variation often move in opposite directions, as a reduction in mortality (required to achieve longer life expectancies) may also lead to a reduction in the variability around the age at death (required to achieve lower lifespan variation). However, this phenomenon is contingent upon mortality improvements in specific age groups (Aburto et al., 2020). In other words, longer lifespans combined with reductions in lifespan variation can be achieved by reducing mortality for midlife adults, while improvements in mortality for older adults may lead to wider lifespan variation. In a previous analysis of temporal trends in longevity and lifespan variation by gender from 1980 to 2016 in the US, van Raalte et al. (2018) found that there have been increases in longevity until 2014, with subsequent declines but also fluctuations in lifespan variation during the 1980s, early 2000s, and mid-2010's, coinciding with mortality increases of young- and middle-aged adults due to HIV/AIDS, the opioid crisis, and the rise in *Deaths of Despair* (Case & Deaton, 2015). Other studies have supported this phenomenon of disproportionate differences in early-life mortality as a key factor in influencing lifespan variation (Hiam et al., 2021; Vaupel et al., 2011; Xu et al., 2021). Our finding of increasing lifespan variation in the last period with concurrent increases, stagnation, and decline of their corresponding life expectancies showcases the complexity of the current changes in mortality by age, as mortality seems to continue improving for older adults while worsening for midlife adults (Harper et al., 2021).

### 4.3 Evidence of rural penalty in life expectancy and lifespan variation

Beyond overall trends, we also found that life expectancy was lower in more rural areas, but this rural penalty gradient varied by racial/ethnic group. These results aligned with Singh et al. (2017) and Singh and Siahpush (2014), who documented an inverse relationship between life expectancy and rurality (i.e., the more rural, the shorter the longevity) and a widening of rural-urban inequalities in longevity from 1969 to 2009. Our findings also aligned with Dwyer-Lindgren et al. (2017), who found substantially growing geographic inequalities in life expectancy. We also documented an increasing gradient in lifespan variation across the continuum of urbanization, in which variability around average age at death increases as counties become more rural (with the exception of metropolitan Black men whose lifespan variations were higher in more urban areas and lower in more rural areas).

Our findings of the rural penalty in longevity and lifespan variation may reflect differences in individual, structural, and contextual determinants of health between metropolitan and nonmetropolitan areas (McCulley et al., 2022). Behaviors such as smoking and lack of leisure-time physical activity are more common among nonmetropolitan residents than metropolitan residents (Eberhardt & Pamuk, 2004; McCulley et al., 2022). Regarding structural and contextual determinants, poverty is higher in nonmetropolitan areas (U.S. Economic Research Service, 2021) and nonmetropolitan areas have lower access to healthcare (Cosby et al., 2008) and lower standards of care (Cossman et al., 2010) that put their residents at higher mortality risks. In addition, mortality improvements in metropolitan areas have consistently outpaced that of nonmetropolitan areas since the 1980s (Cosby et al., 2019), manifested by substantial mortality declines in “more urban” areas (i.e., large central, large fringe) and less favorable trends in “more rural” areas (i.e., micropolitan, noncore) (Graetz & Elo, 2021).

### 4.4 Inequalities by race/ethnicity and gender in the rural penalty in life expectancy and lifespan variation

A key finding was the presence of a “double burden of mortality” (Seaman et al., 2019; van Raalte et al., 2018) for metropolitan and nonmetropolitan Black men and women: shorter (lower life expectancy) and more uncertain (higher lifespan variation) lives. Our findings are consistent with Danaei et al. (2010) and Murray et al. (2006), who both found the widest gap in longevity between Asian/Pacific Islander individuals and Black individuals in high-risk urban areas or in rural South. In addition, Lynch et al. (2003) and Edwards and Tuljapurkar (2005) both found greater variability in age at death for Black than White individuals.

As evidenced in our examination of life expectancy and lifespan variation at birth, 10- 35- and 65-years of age, such high lifespan variations among Black men and women reflect burdens in mortality at young- and middle-ages. Although there is no single cause for the disproportionate midlife mortality among Black individuals in particular, researchers have documented substantial increases in Black midlife mortality from fatal drug overdoses, homicides, followed by alcohol related liver diseases, suicides, and deaths from mental and behavioral disorders (substance and alcohol use disorders) between 1999 and 2016 (Woolf et al., 2018). Additionally, prior studies have shown that diseases tied to behavioral and metabolic risk factors (e.g., cardiovascular diseases, respiratory diseases, diabetes, obesity), and cancers (Danaei et al., 2010; Dwyer-Lindgren et al., 2016, 2017; Murray et al., 2006; Woolf et al., 2018) disproportionately afflict Black individuals and potentially influence their early-life mortality burdens. The “double burden of mortality” among Black men and women may also be the result of other individual- and community-level determinants, specifically historical and current structural racism (Bailey et al., 2021) and racial capitalism (McClure et al., 2020) that creates differential exposure to health-damaging factors and differential vulnerability to their effects (Diderichsen et al., 2019). Following the fundamental causes theory (Link & Phelan, 1995) and life course theory (Niedzwiedz et al., 2012), Black men and women may consequentially lack social and economic resources required to achieve optimal health behaviors due to such factors, leading to shorter lifespans and greater variability with respect to age at death. Nevertheless, the current paucity of evidence between midlife mortality and lifespan variation merits additional research at a granular level to show how these determinants influence the length and variability of lifespans.

### 4.5 Strengths and limitations

This study has several strengths, specifically it is one of the first studies to examine lifespan variation together with life expectancy by race/ethnicity, gender, and urbanicity in the US Although to our knowledge no studies have examined lifespan variation by urbanicity in the US, some have documented it by race/ethnicity (albeit racial groups were limited to only White and Black individuals (Firebaugh et al., 2014; Lynch et al., 2003) or by other demographic factors such as levels of educational attainment (Edwards & Tuljapurkar, 2005; Sasson, 2016) or socioeconomic status (Edwards & Tuljapurkar, 2005). However, we acknowledge some limitations. First, vital registration mortality data and population denominators for race/ethnic groups are sometimes incomplete and/or over- or under-estimated, particularly for Hispanic and Asian/Pacific Islander individuals (Singh & Siahpush, 2014). We excluded a limited number of state-year observations for Hispanics, following NCHS recommendations (National Center for Health Statistics, 2022), but this exclusion only accounted for 0.4–0.8% of the total Hispanic population in the US. Second, we excluded non-Hispanic American Indian/Alaskan Native individuals in our analyses due to issues of misclassification on death certificates (Harper et al., 2021). Third, the census bridged-race classification combines race/ethnic groups that may have differences in mortality (Liebler & Halpern-Manners, 2008), especially Asian individuals and Pacific Islander individuals, that are combined in the same group, obscuring wide heterogeneity (Mau et al., 2009). Fourth, we were not able to examine inequalities in longevity between Hispanic origin subgroups (e.g., Cuban, Mexican, Puerto Rican, Central American), or Asian/Pacific Islanders origin subgroups (e.g., Chinese, Indian, Filipino, Korean) or individuals with multiple race/ethnicities despite known differences in mortality between subgroups (Arias et al., 2020; Guadamuz et al., 2021). Fifth, we acknowledge that temporal trends in lifespan variation differ when utilizing relative lifespan variation measured as coefficient of variation versus lifespan variation as an absolute inequality (standard deviation). Lastly, we employed a single year of the NCHS Urban-Rural Classification Scheme (2013) to classify urbanicity, which may induce measurement error. However, our sensitivity analysis using a different year (1990) showed no appreciable changes in either life expectancy of lifespan variation.

## 5 Conclusion

We documented widening inequalities by race/ethnicity, gender, and urbanicity in life expectancy and lifespan variation from 1990 to 2019. Recent years show a stagnation in life expectancy coupled with a consistent increase in lifespan variation, highlighting uneven mortality patterns in early-life deaths. Black individuals were found to not only live shorter lifespans but also have the most variability with respect to age at death. These findings merit further investigations into underlying structural and contextual determinants as well as overlying health and social policies that lead to the disproportionate burden of premature deaths among such populations. By doing so, these disadvantaged populations can achieve longer lifespans and more homogenous ages at death.

## Author Statement

Conceptualization: I.P.D., A.H.A. and U.B.; Data curation: I.P.D. and U.B.; Formal analysis: I.P.D. and U.B.; Funding acquisition: U.B.; Investigation: I.P.D., A.H.A. and U.B.; Methodology: I.P.D. and U.B.; Project administration: U.B.; Resources: U.B.; Software: I.P.D. and U.B.; Supervision: A.H.A. and U.B.; Validation: I.P.D. and U.B.; Visualization: I.P.D. and U.B.; Writing – original draft: I.P.D. and U.B.; Writing - review & editing: I.P.D., A.H.A. and U.B.;

## Funding

UB was supported by the Office of the Director of the National Institutes of Health under grant number DP5OD26429. The funder had no role in the design of the study, data collection, analysis or interpretation, on the writing of the manuscript or on the decision to submit.

## Ethical statement

This research was deemed exempt under 45 CF 46.104(d)(4)(i) and (ii) by the Drexel University Institutional Review Board.

## Declaration of competing interest

The authors declare no conflict of interest.

## Data Availability

Data cannot be shared (NCHS restricted data). Code is publicly available in a repository (see end of methods section).
