# Supplementary material for: Exploring inequalities in life expectancy and lifespan variation by race/ethnicity and urbanicity in the United States: 1990 to 2019
Source: SSM Popul Health. 2022 Sep 13;19:101230. doi: 10.1016/j.ssmph.2022.101230 (PMC9485214; doi:10.1016/j.ssmph.2022.101230)
Supplement: Multimedia component 1 [file mmc1.docx]

# Appendix

# Appendix Table 1. County boundary changes, 1990-2019.

| **Original FIPS** | **New FIPS Assigned** | **Reason & Solution** |
| --- | --- | --- |
| 51780 | 51083 | In 1995, independent city of South Boston (51780) merged into Halifax county (51083) |
| 12025 | 12086 | In 1997, Dade county (12025) was renamed as Miami-Dade county (12086) |
| 30113 | 30031 | In 1997, Yellowstone Park territory (30113) merged into Gallantin (30031) and Park (30067) counties. Since Gallantin has the largest population, Yellowstone and Park counties were merged into Gallantin. |
| 30067 |  |  |
| 46102 | 46113 | In 2010, Shannon county (46113) was renamed as Oglala Lakota county (46102) |
| 51560 | 51005 | In 2000, Clifton Forge city (51560) was absorbed into Allegheny County (51005) |
| 13999 | Excluded | For years 1990-1991, 13999 represented counties of <100k or HIV deaths (≤ 3) occurring in Georgia. These deaths do not appear in county death rates, but are included in state and national death rates. Therefore, 13999 was excluded from study. |

Footnote: non-continental FIPS were excluded from the study with the exception of Hawaii.

# Appendix Table 2. NCHS 2013 Urban-Rural Classification Scheme.

| Type | Description |
| --- | --- |
| Large central metropolitan county group | Counties in metropolitan statistical areas (MSAs) of ≥1,000,000 population that (1) contain the entire population of the largest principal city of the MSA, or (2) have their entire population contained in the largest principal city of the MSA, or (3) contain ≥250,000 inhabitants of any principal city of the MSA |
| Large fringe metropolitan county group | Counties in MSAs of ≥1,000,000 population that did not qualify as large central metropolitan counties |
| Medium metropolitan county group | Counties in MSAs of populations of 250,000 to 999,999 |
| Small metropolitan county group | Counties in MSAs of populations <250,000 |
| Micropolitan county (nonmetropolitan) group | Counties in micropolitan statistical areas (i.e., urban cluster population of 10,000 to 49,999) |
| Noncore county (nonmetropolitan) group | Nonmetropolitan counties that did not qualify as micropolitan |

# Appendix Table 3. Descriptive table of lifespan variation measures.

| **Lifespan Variation Measure** | **Definition** | **Source** |
| --- | --- | --- |
| Standard deviation & variance | Standard deviation is the average weighted deviations of ages at death from the hypothetical average at death (i.e., life expectancy). Standard deviation can be denoted as $S_{0}$, the variability around life expectancy at birth, $S_{10}$, the variability around remaining life expectancy for individuals aged 10 and older (i.e., conditional upon survival up to age 10), and so on. Variance is the standard deviation squared. | Shkolnikov & Andreev 2010 |
| Coefficient of Variance | The coefficient of variance is the standard deviation of average age at death (i.e., life expectancy) divided by the mean age at death (i.e., life expectancy). | Aburto et al., 2020  Aburto & van Raalte 2018  Edwards & Tuljapurkar 2005  Németh 2017 |
| Gini coefficient | Gini coefficient is related to the Lorenz curve, which in regard to mortality data, is the cumulative share in years of life as a function of share in the lifetable population. The Lorenz curve is perfectly diagonal when everyone has the same length of life. The Gini coefficient is the area between the Lorenz curve and the diagonal, and ranges from 0 (complete equality) to 1 (complete inequality or greater magnitude of inequality in age at death). | Shkolnikov & Andreev 2010  Shkolnikov, Andreev & Begun 2003 |
| Average Inter-Individual Difference (AID) | AID is the average inter-individual difference in life expectancy and captures between-person differences. Measured as years of length of life, AID is the expected difference between life expectancy when two individuals are picked randomly. | Permanyer & Shi 2022  Shkolnikov, Andreev & Begun 2003 |
| Life disparity (e-dagger) | Life disparity, commonly known as $e^{\dagger}$, e-dagger, is the average remaining life expectancy at the age when death occurs. It also reflects “lifetime losses,” or the average life expectancy losses due to death. | Shkolnikov & Andreev 2010  Hiam, Minton & McKee 2021  Vaupel, Zhang & van Raalte 2011 |
| Interquartile range (IQR) | IQR represents the range or span of ages containing the middle 50% of deaths. | Shkolnikov & Andreev 2010  van Raate, Sasson & Martikainen 2018  Wilmoth & Horiuchi 1999 |

Footnote: see references for “source” column at end of appendix

## Appendix Table 4. Selected characteristics for six rural-urban continuum categories, 2015-2019.

| **Characteristic** | **Total** |  | **Metropolitan Counties** | | | | |  | **Non-metropolitan Counties** | | |
| --- | --- | --- | --- | --- | --- | --- | --- | --- | --- | --- | --- |
|  |  |  | **All**  **metro** |  |  |  |  |  | **All non-metro** |  |  |
|  |  |  |  | **Large central** | **Large fringe** | **Medium** | **Small** |  |  | **Micro** | **Noncore** |
| Number of counties, 2015-2019 | 3,141 |  | 1,166 | 68 | 368 | 372 | 358 |  | 1,975 | 641 | 1334 |
| *Percent of total* |  |  | *37%* | *2%* | *12%* | *12%* | *11%* |  | *63%* | *20%* | *42%* |
| Total U.S. population, 2015-2019, in millions | 322.0 |  | 277.0 | 99.6 | 80.8 | 67.4 | 29.2 |  | 45.0 | 26.7 | 18.3 |
| *Percent of total* |  |  | *86%* | *31%* | *25%* | *21%* | *9%* |  | *14%* | *8%* | *6%* |
| Total U.S. deaths, 2015-2019, in millions | 2.8 |  | 2.3 | 0.72 | 0.64 | 0.60 | 0.29 |  | 0.52 | 0.29 | 0.22 |
| *Percent of total* |  |  | *82%* | *26%* | *23%* | *21%* | *10%* |  | *19%* | *10%* | *8%* |
| Non-Hispanic Whites, deaths (%) | 78.2% |  | 75.7% | 60.8% | 82.6% | 80.6% | 87.1% |  | 89.3% | 88.8% | 89.8% |
| Non-Hispanic Blacks, deaths (%) | 12.1% |  | 13.2% | 20.4% | 10.2% | 10.1% | 8.3% |  | 7.4% | 7.1% | 7.9% |
| Non-Hispanic Asian / Pacific Islanders, deaths (%) | 2.6% |  | 3.1% | 5.3% | 2.3% | 2.3% | 0.9% |  | 0.5% | 0.8% | 0.2% |
| Hispanic deaths (%) | 7.1% |  | 8.1% | 13.5% | 4.9% | 7.1% | 3.8% |  | 2.8% | 3.3% | 2.1% |

# Appendix Table 5. Temporal trends life expectancy by race/ethnicity, gender, and urbanicity: United States, 1990-2019.

|  | **Metropolitan Men** | | | | | |
| --- | --- | --- | --- | --- | --- | --- |
| **Race** | **1990-1994** | **1995-1999** | **2000-2004** | **2005-2009** | **2010-2014** | **2015-2019** |
| Overall | 72.3 (72.3, 72.4) | 73.7 (73.7, 73.8) | 74.9 (74.9, 74.9) | 76.1 (76.1, 76.1) | 77.1 (77.1, 77.1) | 77.0 (77.0, 77.1) |
| NHW | 73.3 (73.3, 73.3) | 74.5 (74.5, 74.5) | 75.4 (75.4, 75.4) | 76.4 (76.4, 76.4) | 77.1 (77.0, 77.1) | 76.9 (76.9, 76.9) |
| NHB | 64.3 (64.2, 64.3) | 66.6 (66.5, 66.6) | 68.6 (68.6, 68.6) | 70.3 (70.3, 70.4) | 72.3 (72.3, 72.4) | 72.1 (72.1, 72.1) |
| NHAPI | 79.7 (79.6, 79.8) | 80.8 (80.8, 80.9) | 82.3 (82.3, 82.4) | 83.7 (83.6, 83.7) | 84.8 (84.7, 84.8) | 85.4 (85.4, 85.4) |
| H | 75.6 (75.6, 75.7) | 76.7 (76.7, 76.8) | 77.9 (77.9, 78.0) | 79.3 (79.2, 79.3) | 80.8 (80.7, 80.8) | 80.9 (80.8, 80.9) |
|  | **Metropolitan Women** | | | | | |
| **Race** | **1990-1994** | **1995-1999** | **2000-2004** | **2005-2009** | **2010-2014** | **2015-2019** |
| Overall | 79.2 (79.2, 79.2) | 79.6 (79.6, 79.6) | 80.0 (80.0, 80.1) | 81.1 (81.1, 81.1) | 81.9 (81.9, 81.9) | 82.0 (82.0, 82.0) |
| NHW | 79.8 (79.7, 79.8) | 80.0 (80.0, 80.0) | 80.3 (80.3, 80.3) | 81.1 (81.1, 81.2) | 81.7 (81.7, 81.7) | 81.7 (81.7, 81.7) |
| NHB | 73.8 (73.8, 73.9) | 74.6 (74.6, 74.7) | 75.5 (75.4, 75.5) | 77.1 (77.0, 77.1) | 78.6 (78.6, 78.6) | 78.9 (78.9, 78.9) |
| NHAPI | 85.6 (85.6, 85.7) | 86.5 (86.4, 86.6) | 87.4 (87.3, 87.4) | 88.4 (88.3, 88.4) | 89.3 (89.3, 89.4) | 89.8 (89.7, 89.8) |
| H | 84.0 (83.9, 84.0) | 83.5 (83.4, 83.5) | 83.7 (83.6, 83.7) | 84.8 (84.8, 84.8) | 85.9 (85.9, 85.9) | 86.3 (86.3, 86.3) |
|  | **Nonmetropolitan Men** | | | | | |
| **Race** | **1990-1994** | **1995-1999** | **2000-2004** | **2005-2009** | **2010-2014** | **2015-2019** |
| Overall | 72.0 (72.0, 72.0) | 72.8 (72.7, 72.8) | 73.6 (73.5, 73.6) | 74.3 (74.3, 74.4) | 74.9 (74.8, 74.9) | 74.6 (74.6, 74.6) |
| NHW | 72.5 (72.5, 72.6) | 73.3 (73.2, 73.3) | 73.9 (73.9, 73.9) | 74.5 (74.5, 74.6) | 74.9 (74.9, 74.9) | 74.6 (74.6, 74.6) |
| NHB | 65.3 (65.2, 65.4) | 66.6 (66.5, 66.7) | 68.3 (68.2, 68.4) | 69.5 (69.4, 69.6) | 71.1 (71.0, 71.2) | 70.8 (70.7, 70.9) |
| NHAPI | 76.1 (75.7, 76.6) | 75.8 (75.4, 76.2) | 79.7 (79.3, 80.0) | 80.6 (80.3, 80.9) | 82.3 (82.0, 82.6) | 83.1 (82.8, 83.4) |
| H | 73.9 (73.7, 74.1) | 74.6 (74.4, 74.7) | 76.6 (76.5, 76.8) | 78.3 (78.1, 78.4) | 79.4 (79.3, 79.6) | 79.9 (79.8, 80.0) |
|  | **Nonmetropolitan Women** | | | | | |
| **Race** | **1990-1994** | **1995-1999** | **2000-2004** | **2005-2009** | **2010-2014** | **2015-2019** |
| Overall | 79.1 (79.0, 79.1) | 79.1 (79.0, 79.1) | 79.1 (79.1, 79.1) | 79.6 (79.6, 79.6) | 79.8 (79.8, 79.9) | 79.6 (79.6, 79.6) |
| NHW | 79.5 (79.5, 79.5) | 79.5 (79.4, 79.5) | 79.4 (79.4, 79.4) | 79.8 (79.8, 79.8) | 79.9 (79.9, 79.9) | 79.6 (79.6, 79.6) |
| NHB | 74.0 (73.9, 74.1) | 74.2 (74.1, 74.3) | 74.6 (74.5, 74.7) | 75.6 (75.5, 75.7) | 76.8 (76.7, 76.9) | 76.9 (76.8, 77.0) |
| NHAPI | 81.4 (81.0, 81.8) | 81.2 (80.8, 81.6) | 85.4 (85.1, 85.8) | 85.9 (85.5, 86.2) | 87.3 (87.0, 87.5) | 87.7 (87.4, 87.9) |
| H | 80.9 (80.7, 81.0) | 80.8 (80.7, 81.0) | 82.3 (82.2, 82.5) | 83.3 (83.1, 83.4) | 84.1 (83.9, 84.2) | 84.8 (84.7, 84.9) |

# Appendix Table 6. Life expectancy by race/ethnicity, gender, by levels of urbanization: United States, 2015-2019.

|  | **Men** | | | | | | | |
| --- | --- | --- | --- | --- | --- | --- | --- | --- |
| **Race** | ***All metro***  ***counties*** | **Large central** | **Large fringe** | **Medium** | **Small** | ***All nonmetro***  ***counties*** | **Micro** | **Noncore** |
| Overall | 77.0 (77.0, 77.1) | 77.5 (77.5, 77.5) | 77.7 (77.7, 77.7) | 76.2 (76.2, 76.2) | 75.7 (75.6, 75.7) | 74.6 (74.6, 74.6) | 74.7 (74.7, 74.8) | 74.3 (74.3, 74.4) |
| NHW | 76.9 (76.9, 76.9) | 77.6 (77.6, 77.6) | 77.3 (77.3, 77.4) | 76.2 (76.2, 76.3) | 75.7 (75.7, 75.8) | 74.6 (74.6, 74.6) | 74.7 (74.7, 74.8) | 74.3 (74.3, 74.4) |
| NHB | 72.1 (72.1, 72.1) | 71.3 (71.3, 71.4) | 74.4 (74.3, 74.5) | 71.4 (71.3, 71.5) | 71.1 (71.0, 71.3) | 70.8 (70.7, 70.9) | 70.6 (70.5, 70.8) | 70.9 (70.8, 71.1) |
| NHAPI | 85.4 (85.4, 85.4) | 85.4 (85.4, 85.5) | 87.1 (87.0, 87.2) | 83.0 (82.9, 83.2) | 84.8 (84.6, 85.1) | 83.1 (82.8, 83.4) | 82.2 (81.8, 82.5) | 89.7 (88.9, 90.5) |
| H | 80.9 (80.8, 80.9) | 80.8 (80.7, 80.8) | 82.9 (82.8, 82.9) | 79.5 (79.5, 79.6) | 80.5 (80.3, 80.6) | 79.9 (79.8, 80.0) | 79.3 (79.2, 79.5) | 81.1 (80.9, 81.3) |
|  | **Women** | | | | | | | |
| **Race** | ***All metro***  ***counties*** | **Large central** | **Large fringe** | **Medium** | **Small** | ***All nonmetro***  ***counties*** | **Micro** | **Noncore** |
| Overall | 82.0 (82.0, 82.0) | 82.8 (82.7, 82.8) | 82.4 (82.4, 82.4) | 81.3 (81.3, 81.3) | 80.7 (80.6, 80.7) | 79.6 (79.6, 79.6) | 79.7 (79.7, 79.8) | 79.4 (79.4, 79.5) |
| NHW | 81.7 (81.7, 81.7) | 82.3 (82.3, 82.4) | 82.0 (82.0, 82.0) | 81.1 (81.1, 81.1) | 80.7 (80.6, 80.7) | 79.6 (79.6, 79.6) | 79.7 (79.6, 79.7) | 79.5 (79.4, 79.5) |
| NHB | 78.9 (78.9, 78.9) | 78.7 (78.7, 78.7) | 80.3 (80.3, 80.4) | 78.0 (77.9, 78.1) | 77.4 (77.3, 77.5) | 76.9 (76.8, 77.0) | 76.7 (76.5, 76.8) | 77.1 (76.9, 77.2) |
| NHAPI | 89.8 (89.7, 89.8) | 90.0 (90.0, 90.1) | 90.8 (90.7, 90.9) | 87.9 (87.8, 88.0) | 88.8 (88.6, 89.1) | 87.7 (87.4, 87.9) | 87.1 (86.8, 87.4) | 90.7 (90.1, 91.3) |
| H | 86.3 (86.3, 86.3) | 86.5 (86.4, 86.5) | 87.6 (87.5, 87.6) | 84.8 (84.8, 84.9) | 85.9 (85.8, 86.0) | 84.8 (84.7, 84.9) | 84.5 (84.3, 84.6) | 85.5 (85.3, 85.7) |

# Appendix Table 7. Temporal trends in metropolitan lifespan variation (coefficient of variation) by race/ethnicity, gender, and urbanicity: United States, 1990-2019.

|  | **Metropolitan Men** | | | | | |
| --- | --- | --- | --- | --- | --- | --- |
| **Race** | **1990-1994** | **1995-1999** | **2000-2004** | **2005-2009** | **2010-2014** | **2015-2019** |
| Overall | 0.247 | 0.233 | 0.227 | 0.226 | 0.219 | 0.225 |
| NHW | 0.230 | 0.220 | 0.217 | 0.219 | 0.215 | 0.221 |
| NHB | 0.322 | 0.298 | 0.281 | 0.277 | 0.264 | 0.272 |
| NHAPI | 0.200 | 0.191 | 0.184 | 0.181 | 0.179 | 0.186 |
| H | 0.254 | 0.231 | 0.221 | 0.217 | 0.207 | 0.214 |
|  | **Metropolitan Women** | | | | | |
| **Race** | **1990-1994** | **1995-1999** | **2000-2004** | **2005-2009** | **2010-2014** | **2015-2019** |
| Overall | 0.204 | 0.197 | 0.192 | 0.190 | 0.185 | 0.187 |
| NHW | 0.192 | 0.188 | 0.185 | 0.185 | 0.182 | 0.184 |
| NHB | 0.263 | 0.251 | 0.239 | 0.233 | 0.224 | 0.227 |
| NHAPI | 0.175 | 0.171 | 0.164 | 0.158 | 0.155 | 0.156 |
| H | 0.198 | 0.187 | 0.179 | 0.176 | 0.170 | 0.173 |
|  | **Nonmetropolitan Men** | | | | | |
| **Race** | **1990-1994** | **1995-1999** | **2000-2004** | **2005-2009** | **2010-2014** | **2015-2019** |
| Overall | 0.247 | 0.240 | 0.238 | 0.238 | 0.235 | 0.240 |
| NHW | 0.239 | 0.234 | 0.234 | 0.236 | 0.235 | 0.240 |
| NHB | 0.302 | 0.289 | 0.273 | 0.269 | 0.259 | 0.268 |
| NHAPI | 0.227 | 0.220 | 0.204 | 0.203 | 0.198 | 0.213 |
| H | 0.247 | 0.235 | 0.230 | 0.227 | 0.222 | 0.228 |
|  | **Nonmetropolitan Women** | | | | | |
| **Race** | **1990-1994** | **1995-1999** | **2000-2004** | **2005-2009** | **2010-2014** | **2015-2019** |
| Overall | 0.207 | 0.204 | 0.202 | 0.202 | 0.202 | 0.206 |
| NHW | 0.201 | 0.198 | 0.197 | 0.198 | 0.200 | 0.205 |
| NHB | 0.263 | 0.257 | 0.249 | 0.244 | 0.235 | 0.242 |
| NHAPI | 0.193 | 0.201 | 0.185 | 0.182 | 0.174 | 0.179 |
| H | 0.200 | 0.196 | 0.190 | 0.187 | 0.183 | 0.189 |

# Appendix Table 8. Lifespan variation by race/ethnicity, gender, and levels of urbanization: United States, 2015-2019.

|  | **Men** | | | | | | | |
| --- | --- | --- | --- | --- | --- | --- | --- | --- |
| **Race** | ***All metro***  ***counties*** | **Large central** | **Large fringe** | **Medium** | **Small** | ***All nonmetro counties*** | **Micro** | **Noncore** |
| Overall | 0.225 | 0.223 | 0.218 | 0.232 | 0.233 | 0.240 | 0.238 | 0.244 |
| NHW | 0.221 | 0.212 | 0.218 | 0.229 | 0.230 | 0.240 | 0.237 | 0.244 |
| NHB | 0.272 | 0.281 | 0.255 | 0.275 | 0.272 | 0.268 | 0.270 | 0.266 |
| NHAPI | 0.186 | 0.184 | 0.180 | 0.201 | 0.201 | 0.213 | 0.215 | 0.214 |
| H | 0.214 | 0.212 | 0.208 | 0.221 | 0.221 | 0.228 | 0.226 | 0.234 |
|  | **Women** | | | | | | | |
| **Race** | ***All metro***  ***counties*** | **Large central** | **Large fringe** | **Medium** | **Small** | ***All nonmetro counties*** | **Micro** | **Noncore** |
| Overall | 0.187 | 0.184 | 0.181 | 0.194 | 0.197 | 0.206 | 0.204 | 0.210 |
| NHW | 0.184 | 0.177 | 0.181 | 0.192 | 0.194 | 0.205 | 0.202 | 0.208 |
| NHB | 0.227 | 0.231 | 0.214 | 0.233 | 0.236 | 0.242 | 0.240 | 0.244 |
| NHAPI | 0.156 | 0.155 | 0.152 | 0.167 | 0.167 | 0.179 | 0.181 | 0.176 |
| H | 0.173 | 0.171 | 0.168 | 0.180 | 0.183 | 0.189 | 0.186 | 0.194 |

Appendix Table 9: Life expectancy at birth (e_0_) and at ages 10 (e_10_), 35 (e_35_), and 65 (e_65_) and lifespan variation as coefficient of variance at birth (CV_0_) and at ages 10 (CV_10_), 35 (CV_35_), and 65 (CV_65_)for metropolitan racial groups by gender: United States, 2015-2019.

|  | **Metropolitan Men** | | | | | | | |
| --- | --- | --- | --- | --- | --- | --- | --- | --- |
| **Race** | e_0_ | CV_0_ | e_10_ | CV_10_ | e_35_ | CV_35_ | **e(65)** | CV_65_ |
| Overall | 77.0 (77.0, 77.1) | 0.225 | 67.6 (67.6, 67.6) | 0.236 | 44.1 (44.1, 44.1) | 0.308 | 18.8 (18.8, 18.8) | 0.523 |
| NHW | 76.9 (76.9, 76.9) | 0.221 | 67.4 (67.4, 67.4) | 0.235 | 43.8 (43.8, 43.8) | 0.308 | 18.6 (18.6, 18.6) | 0.519 |
| NHB | 72.1 (72.1, 72.1) | 0.272 | 63.2 (63.2, 63.2) | 0.278 | 40.4 (40.3, 40.4) | 0.364 | 17.0 (17.0, 17.0) | 0.673 |
| NHAPI | 85.4 (85.3, 85.4) | 0.186 | 75.9 (75.8, 75.9) | 0.191 | 51.6 (51.5, 51.6) | 0.253 | 24.3 (24.3, 24.3) | 0.402 |
| H | 80.9 (80.8, 80.9) | 0.214 | 71.4 (71.3, 71.4) | 0.225 | 47.6 (47.6, 47.6) | 0.291 | 21.5 (21.5, 21.6) | 0.473 |
|  | **Metropolitan Women** | | | | | | | |
| **Race** | e_0_ | CV_0_ | e_10_ | CV_10_ | e_35_ | CV_35_ | **e(65)** | CV_65_ |
| Overall | 82.0 (82.0, 82.0) | 0.187 | 72.6 (72.5, 72.6) | 0.191 | 48.2 (48.2, 48.2) | 0.260 | 21.4 (21.4, 21.4) | 0.415 |
| NHW | 81.7 (81.7, 81.7) | 0.184 | 72.1 (72.1, 72.1) | 0.193 | 47.8 (47.8, 47.8) | 0.260 | 21.0 (21.0, 21.0) | 0.415 |
| NHB | 78.9 (78.9, 78.9) | 0.227 | 69.9 (69.8, 69.9) | 0.224 | 45.8 (45.7, 45.8) | 0.309 | 20.4 (20.4, 20.5) | 0.504 |
| NHAPI | 89.8 (89.7, 89.8) | 0.156 | 80.2 (80.2, 80.2) | 0.157 | 55.5 (55.5, 55.6) | 0.211 | 27.2 (27.2, 27.3) | 0.330 |
| H | 86.3 (86.3, 86.3) | 0.173 | 76.7 (76.7, 76.8) | 0.177 | 52.3 (52.2, 52.3) | 0.237 | 24.6 (24.6, 24.6) | 0.376 |
|  | **Nonmetropolitan Men** | | | | | | | |
| **Race** | e_0_ | CV_0_ | e_10_ | CV_10_ | e_35_ | CV_35_ | **e(65)** | CV_65_ |
| Overall | 74.6 (74.6, 74.6) | 0.240 | 65.3 (65.2, 65.3) | 0.252 | 41.9 (41.9, 41.9) | 0.333 | 17.6 (17.6, 17.6) | 0.582 |
| NHW | 74.6 (74.6, 74.6) | 0.240 | 65.2 (65.2, 65.3) | 0.253 | 41.9 (41.9, 41.9) | 0.332 | 17.6 (17.6, 17.6) | 0.579 |
| NHB | 70.8 (70.7, 70.9) | 0.268 | 61.9 (61.8, 62.0) | 0.272 | 38.7 (38.6, 38.7) | 0.373 | 15.9 (15.8, 15.9) | 0.707 |
| NHAPI | 83.1 (82.9, 83.4) | 0.213 | 73.9 (73.6, 74.1) | 0.216 | 49.8 (49.6, 50.0) | 0.286 | 23.7 (23.4, 23.9) | 0.437 |
| H | 79.9 (79.8, 80.0) | 0.228 | 70.5 (70.4, 70.6) | 0.240 | 46.9 (46.8, 47.0) | 0.311 | 21.4 (21.3, 21.5) | 0.508 |
|  | **Nonmetropolitan Women** | | | | | | | |
| **Race** | e_0_ | CV_0_ | e_10_ | CV_10_ | e_35_ | CV_35_ | **e(65)** | CV_65_ |
| Overall | 79.6 (79.6, 79.6) | 0.206 | 70.2 (70.2, 70.2) | 0.212 | 46.1 (46.1, 46.1) | 0.288 | 20.2 (20.2, 20.2) | 0.462 |
| NHW | 79.6 (79.6, 79.6) | 0.205 | 70.2 (70.1, 70.2) | 0.212 | 46.1 (46.0, 46.1) | 0.287 | 20.1 (20.1, 20.1) | 0.460 |
| NHB | 76.9 (76.8, 77.0) | 0.242 | 67.9 (67.8, 68.0) | 0.242 | 44.0 (43.9, 44.1) | 0.334 | 19.6 (19.5, 19.7) | 0.545 |
| NHAPI | 87.7 (87.4, 87.9) | 0.179 | 78.2 (78.0, 78.4) | 0.182 | 53.8 (53.5, 54.0) | 0.240 | 26.0 (25.8, 26.2) | 0.379 |
| H | 84.8 (84.7, 84.9) | 0.189 | 75.3 (75.2, 75.4) | 0.194 | 51.0 (50.9, 51.1) | 0.259 | 23.9 (23.8, 24.0) | 0.412 |

**Appendix Figure 1**. Pearson correlation coefficients between pairs of measures of lifespan variation based on graduated single year mortality data obtained from the NCHS and population estimates obtained from the U.S. Census Bureau, 2015-2019.

#

## Footnote: See Appendix Table 3 for definitions of lifespan variation measures. Each dot represents one of the 16 race/ethnic, gender, and urbanicity combinations.

## Appendix Figure 2. Association between life expectancy and lifespan variation (measured as standard deviation) by race/ethnicity, gender, and urbanicity: United States, 1990-2019.

**
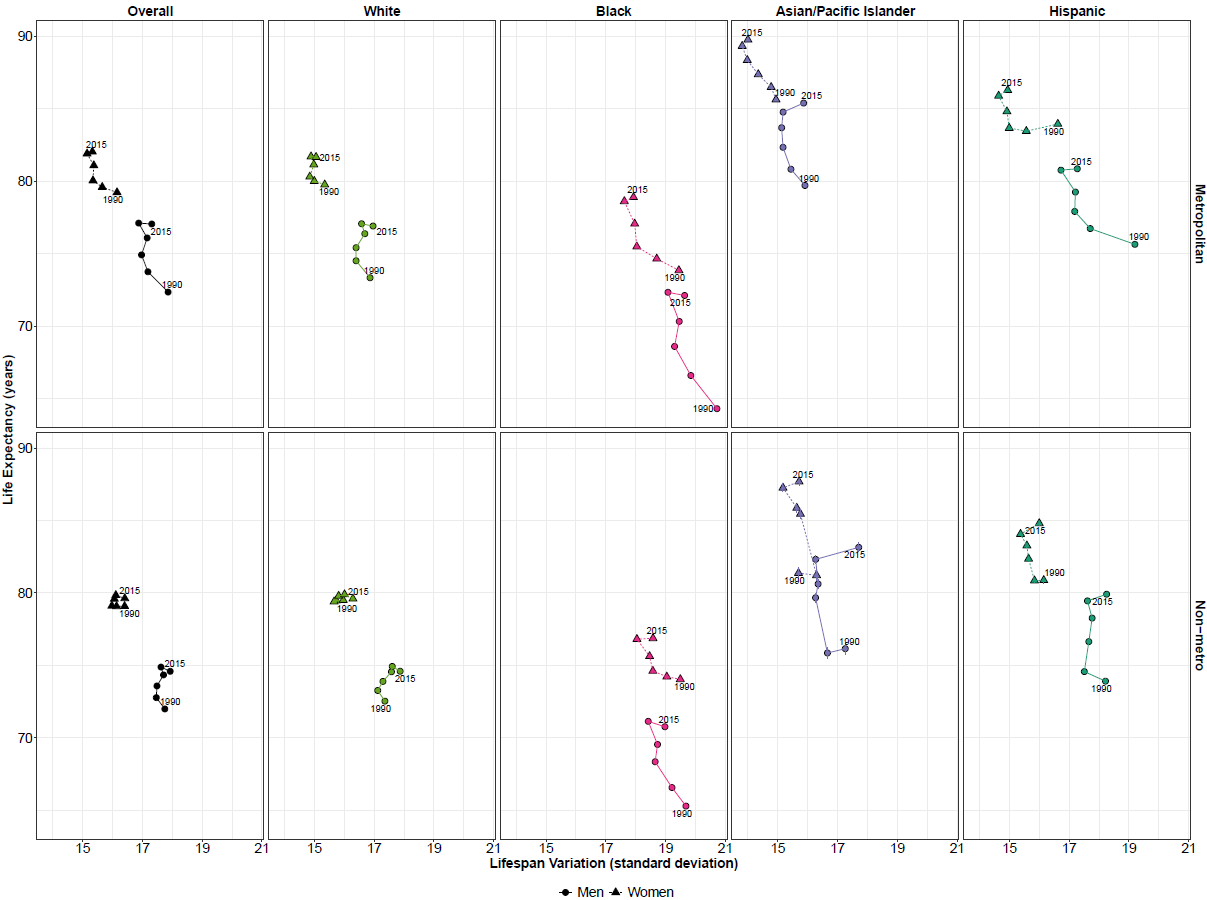
**

**Appendix Figure 3.** Association between relative (coefficient of variation) and absolute (standard deviation) lifespan variation by race/ethnicity, gender, and urbanicity: United States, 1990-2019.

## Appendix Figure 4. Comparison of life expectancy using the 1990 vs 2013 NCHS Urban-Rural Classification Scheme: United States, 2015-2019.


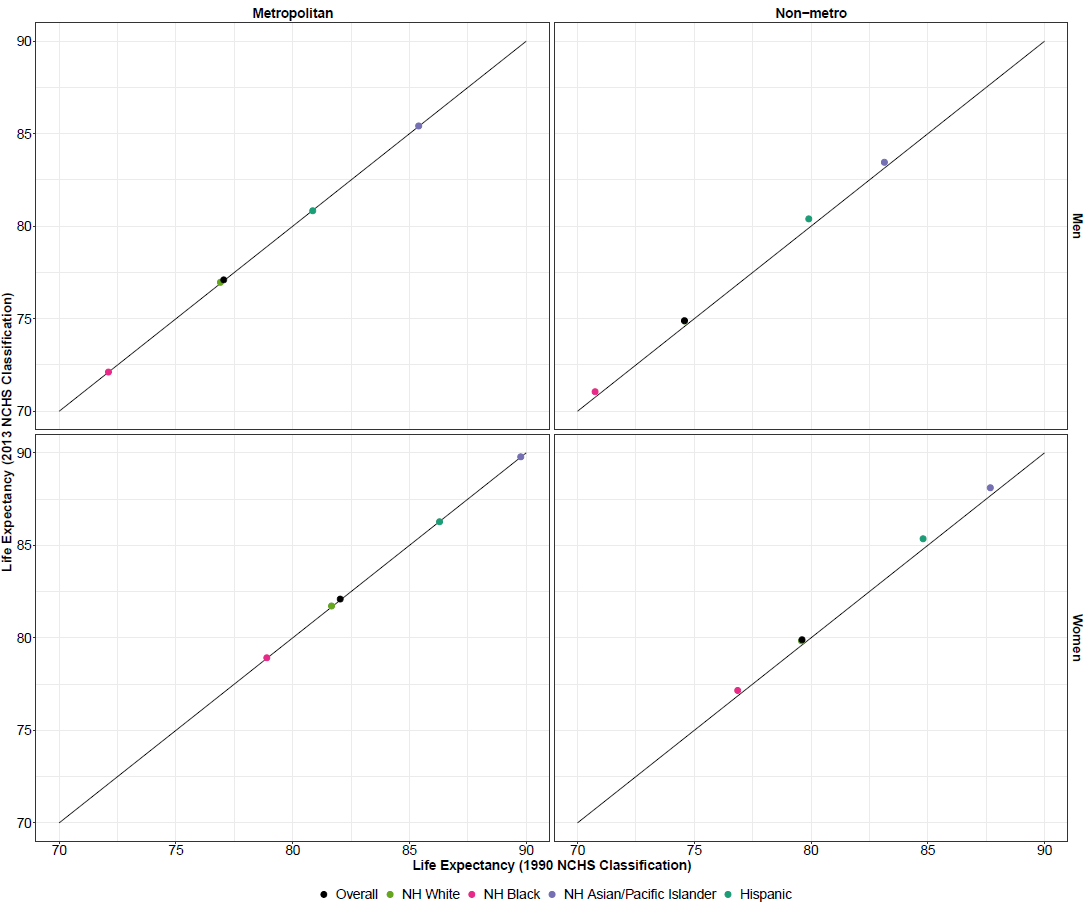


## Appendix Figure 5. Comparison of relative lifespan variation (coefficient of variation) using the 1990 vs 2013 NCHS Urban-Rural Classification Scheme: United States, 2015-2019.


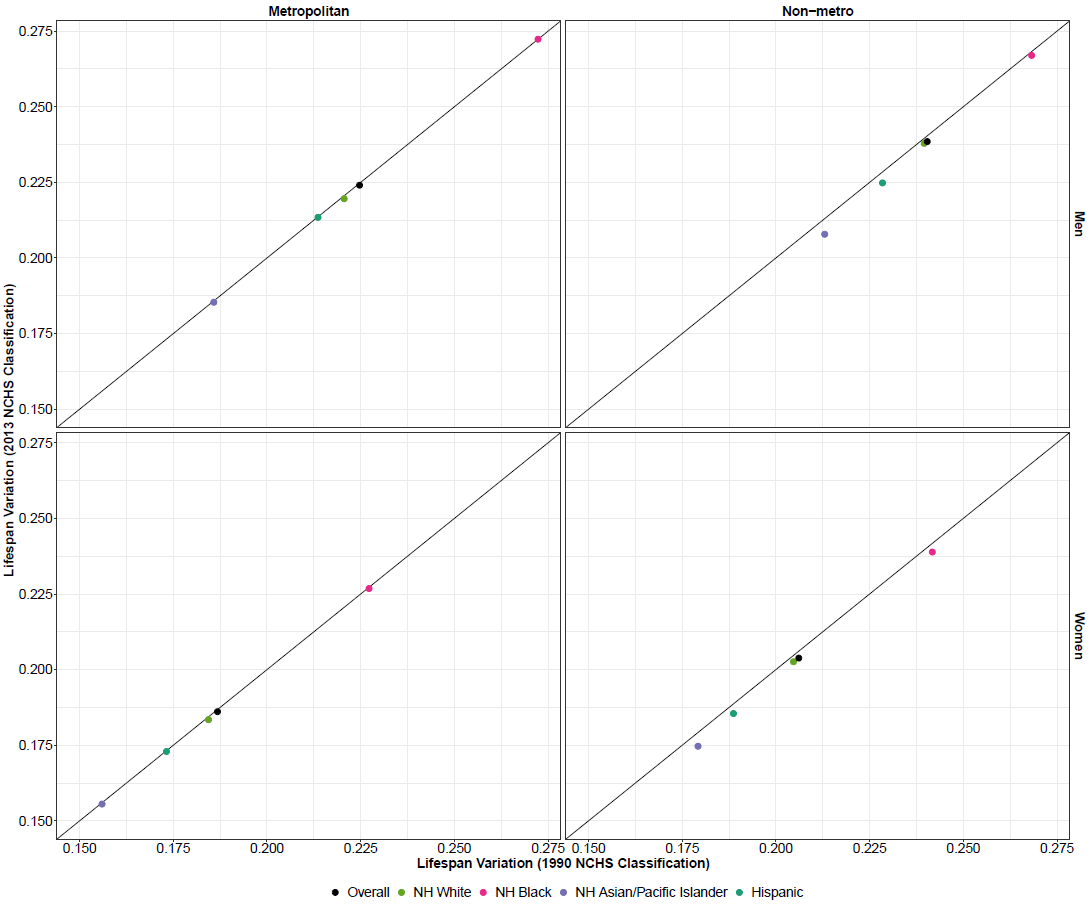


**References**

Aburto, J. M., & van Raalte, A. (2018). Lifespan Dispersion in Times of Life Expectancy Fluctuation: The Case of Central and Eastern Europe. *Demography*, *55*(6), 2071–2096. <https://doi.org/10.1007/s13524-018-0729-9>

Aburto, J. M., Villavicencio, F., Basellini, U., Kjærgaard, S., & Vaupel, J. W. (2020). Dynamics of life expectancy and life span equality. *Proceedings of the National Academy of Sciences of the United States of America*, *117*(10), 5250–5259. <https://doi.org/10.1073/pnas.1915884117>

Edwards, R. D., & Tuljapurkar, S. (2005). Inequality in life spans and a new perspective on mortality convergence across industrialized countries. *Population and Development Review*, *31*(4), 645-674.

Hiam, L., Minton, J., & McKee, M. (2021). What can lifespan variation reveal that life expectancy hides? Comparison of five high-income countries. *J R Soc Med, 114*(8), 389-399. doi:10.1177/01410768211011742

Németh L. (2017). Life expectancy versus lifespan inequality: A smudge or a clear relationship?. *PloS one*, *12*(9), e0185702. https://doi.org/10.1371/journal.pone.0185702

Permanyer, I., & Shi, J. (2022). Normalized lifespan inequality: disentangling the longevity-lifespan variability nexus. *Genus, 78*(1), 2. doi:10.1186/s41118-021-00150-6

van Raalte, A. A., Sasson, I., & Martikainen, P. (2018). The case for monitoring life-span inequality. *Science, 362*(6418), 1002-1004. doi:10.1126/science.aau5811

Shkolnikov, V. M., Andreev, E.M., Begun, A.Z. (2003). Gini coefficient as a life table function: Computation from discrete data, decomposition of differences and empirical examples. *Demographic Research, 8*(11), 305-358.

Shkolnikov, V. M., Andreev, E.M. (2010). *Spreadsheet for calculation of life-table dispersion measures*. Available from: <https://www.demogr.mpg.de/papers/technicalreports/tr-2010-001.pdf>

Vaupel, J. W., Zhang, Z., & van Raalte, A. A. (2011). Life expectancy and disparity: an international comparison of life table data. *BMJ Open, 1*(1), e000128. doi:10.1136/bmjopen-2011-000128

Wilmoth, J. R., & Horiuchi, S. (1999). Rectangularization revisited: variability of age at death within human populations. *Demography, 36*(4), 475-495.
